# The canonical Wg signaling modulates Bsk-mediated cell death in *Drosophila*

**DOI:** 10.1038/cddis.2015.85

**Published:** 2015-04-09

**Authors:** S Zhang, C Chen, C Wu, Y Yang, W Li, L Xue

**Affiliations:** 1 Institute of Intervention Vessel, Shanghai 10th People's Hospital, Shanghai Key Laboratory of Signaling and Disease Research, School of Life Science and Technology, Tongji University, 1239 Siping Road, Shanghai 200092, PR China

## Abstract

Cell death is an essential regulatory mechanism for removing unneeded cells in animal development and tissue homeostasis. The c-Jun N-terminal kinase (JNK) pathway has pivotal roles in the regulation of cell death in response to various intrinsic and extrinsic stress signals. The canonical Wingless (Wg) signaling has been implicated in cell proliferation and cell fate decisions, whereas its role in cell death remains largely elusive. Here, we report that activated Bsk (the *Drosophila* JNK homolog) induced cell death is mediated by the canonical Wg signaling. First, loss of Wg signaling abrogates Bsk-mediated caspase-independent cell death. Second, activation of Wg signaling promotes cell death in a caspase-independent manner. Third, activation of Bsk signaling results in upregulated transcription of *wingles*s *(wg)* gene. Finally, Wg pathway participates in the physiological function of Bsk signaling in development. These findings not only reveal a previously undiscovered role of Wg signaling in Bsk-mediated cell death, but also provide a novel mechanism for the interplay between the two important signaling pathways in development.

Cell death plays a fundamental role in animal development and tissue homeostasis by modulating numerous physiological processes, including regulation of cell number and organ size, sculpting of tissue structures, and elimination of abnormal or aged cells.^1, 2^ Deregulation of cell death would lead to abnormal development and various diseases, such as tumors, neurodegenerative disorders, and immunodeficiency diseases.^3, 4^

c-Jun N-terminal kinase (JNK) signaling is a crucial regulatory mechanism that controls stress-induced cell death. This pathway is initiated by various intrinsic and extrinsic signals including the tumor necrosis factor (TNF) family ligands, and is mediated through a mitogen-activated protein (MAP) kinase cascade.^5^ Upon phosphorylation, JNK translocates into the nucleus and activates the transcription factors Jun, Fos and FoxO, which will finally induce cell death.^6, 7, 8, 9^ JNK pathway is highly conserved in *Drosophila melanogaster*, in which the TNF orthlog Eiger (Egr) triggers cell death through the JNK kinase kinase dTAK1, the JNK kinase Hemipterous (Hep), and *Drosophila* JNK Basket (Bsk).^10, 11, 12, 13, 14^ Although much progress has been made for understanding the core regulatory machine of Egr-Bsk pathway, other signaling pathways that modulate Bsk-mediated cell death have not been fully elucidated.

Wnt proteins are a family of highly conserved secreted molecules that regulate cell–cell interactions through distinct intracellular signal transduction pathways, including the canonical Wnt/*β*-catenin pathway, the Wnt/Ca^2+^ pathway, and the Wnt/Frizzled (Fz)-Planar Cell Polarity (PCP) pathway.^15^ The canonical Wnt signaling represents one of the best investigated pathways and has crucial roles in regulating cell proliferation, cell migration, and cell fate decisions.^16, 17^ Deregulated Wnt signaling is frequently associated with human diseases, especially cancers.^18, 19, 20^ Genetic studies of the *Drosophila* Wnt protein Wingless (Wg) have made great contributions to our understanding of Wnt signaling.^21, 22, 23^ In the absence of Wg signal, the *β*-catenin homolog Armadillo (Arm) is phosphorylated and degraded by a multi-protein 'destruction complex' that includes the Adenomatous Polyposis Coli protein (APC), Shaggy (Sgg)/Zeste-white-3, Axin (Axn), and Casein Kinase1 (CK1). In the presence of Wg signal, the receptor dFrizzled (dFz) and co-receptor Arrow (Arr) bind to Wg, recruit and phosphorylate the scaffold protein Dishevelled (Dsh), which antagonizes the 'destruction complex' and prevents Arm from degradation. As a consequence, accumulated Arm translocates to the nucleus and stimulates target genes expression by binding to the transcription factor Pangolin (Pan).^15, 24, 25^

Crosstalk between different signaling pathways occurs extensively and forms a complex signaling network that enable cells to interpret multiple inputs and execute different responses in a context-dependent manner. The interplay between JNK and the non-canonical Wnt/Frizzled (Fz)-PCP pathways has been extensively studied, in which JNK signaling acts downstream of Dsh to promote PCP establishment.^26, 27, 28^ Yet little is known about the interaction between JNK and the canonical Wnt/Wg pathways in development. A collaboration between JNK and the canonical Wg signaling was reported to promote dorsal closure and ventral patterning during *Drosophila* embryogenesis, but the two pathways appear to function in parallel.^29^ An interaction between AP-1 and *β*-catenin/TCF complex was also reported to have a role in carcinogenesis, though the underlying mechanism remains elusive.^30^

In the present study, we have identified from a genetic screen that the canonical Wg signaling has a crucial role in Bsk-mediated cell death in *Drosophila*. Loss of Wg signaling specifically suppresses Egr or Hep-induced Bsk-dependent cell death in eye, wing, and thorax. Stimulation of Wg signaling phenocopies Bsk activation and induces caspase-independent cell death. Furthermore, Wg signaling is both necessary and sufficient for the expression of Bsk signaling reporter *puckered (puc)*. Epistasis experiments indicate that Wg signaling acts downstream of Bsk to regulate cell death and other physiological functions. Finally, activated Bsk signaling upregulates *wg* transcription, providing a molecular mechanism for the genetic interaction between Bsk and Wg pathways. These findings not only expand our existing knowledge about the molecular basis of complex signaling network in regulating cellular activities, but also provide additional strategy and approach for cancer therapy.

## Results

### Loss of Wg signaling suppresses Egr-induced small eye phenotype in *Drosophila*

**Figure 1:**
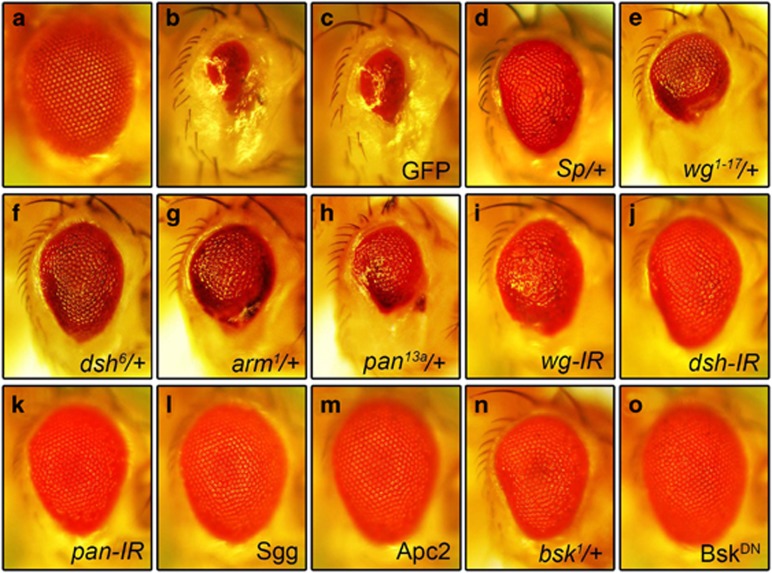
Loss of Wg signaling suppresses Egr-induced small eye phenotype. Light micrographs of *Drosophila* adult eyes are shown. Compared with the GMR-Gal4 control (**a**), *GMR>*Egr triggers extensive cell death and produces a small eye phenotype (**b**), which remains unaffected by expression of GFP (**c**), but is suppressed by *Sp* (**d**) or mutations in *wg* (**e**), *dsh* (**f**), *arm* (**g**), or *pan* (**h**), or RNAi knocking-down of *wg* (**i**), *dsh* (**j**), or *pan* (**k**), or overexpression of Sgg (**l**) or Apc2 (**m**). Mutation in *bsk* (**n**) and expression of Bsk^DN^ (**o**) serve as positive controls

Ectopic expression of TNF ortholog Egr in the developing eye driven by *GMR*-Gal4 (*GMR*>Egr) triggers massive cell death and produces a Bsk-dependent small eye phenotype (Figures 1b, n and o),^10, 11, 31^ as compared with the *GMR*-Gal4 control (Figure 1a). To identify additional genes that modulate Egr-induced cell death, we performed a genetic screen for dominant modifiers of the *GMR*>Egr induced small eye phenotype.^14, 32^ Intriguingly, we found that this phenotype was strongly suppressed in heterozygous *Sternopleural* (*Sp*) animals (Figure 1d). *Sp* was originally identified as a dominant mutation that produces supernumerary sternopleural bristles,^33^ and was later characterized as a regulatory mutation of the *wg* gene.^34^ Consistent with this observation, the *GMR*>Egr induced small eye phenotype is also suppressed by two other *wg* mutant alleles in heterozygosity (Figure 1e and Supplementary Figure S1b) or by the expression of a *wg* RNAi (Figure 1i). Since *wg* encodes a ligand for the Wg signaling pathway, we wondered whether the canonical Wg pathway is required. Indeed, we found that the *GMR*>Egr induced small eye phenotype was suppressed mildly by mutation in *dsh* (Figure 1f and Supplementary Figure S1c), *arm* (Figure 1g and Supplementary Figure S1d), or *pan* (Figure 1h), and strongly by RNAi-mediated knocking-down of *dsh* (Figure 1j) or *pan* (Figure 1k). Furthermore, expression of Sgg or Apc2, components of the 'destruction complex' that negatively regulates Wg signaling, suppressed *GMR*>Egr induced small eye phenotype to a similar extent as that of Bsk^DN^ (Figures 1l, m and o), a dominant-negative form of Bsk encoding the *Drosophila* JNK ortholog. As a negative control, *GMR*>Egr induced small eye phenotype is not affected by the expression of GFP (Figure 1c). Together, these results suggest that the canonical Wg pathway is required for Egr-induced small eye phenotype.

To examine whether Wg pathway is specifically involved in Egr-triggered cell death, we checked whether loss of Wg signaling could affect cell death triggered by *Dp53*, a pro-apoptotic gene encoding the *Drosophila* ortholog of p53.^35, 36, 37^ Ectopic expression of Dp53 in the eye (*GMR*>Dp53) induces vast cell death and produces small and rough eyes with fused ommatidia (Supplementary Figure S2a).^35^ However, this phenotype is not suppressed by downregulation of Wg signaling (Supplementary Figure S2), suggesting that the canonical Wg pathway is specifically required for Egr induced small eye phenotype *in vivo*.

### Loss of Wg signaling suppresses Hep-induced Bsk-mediated small eye phenotype

**Figure 2:**
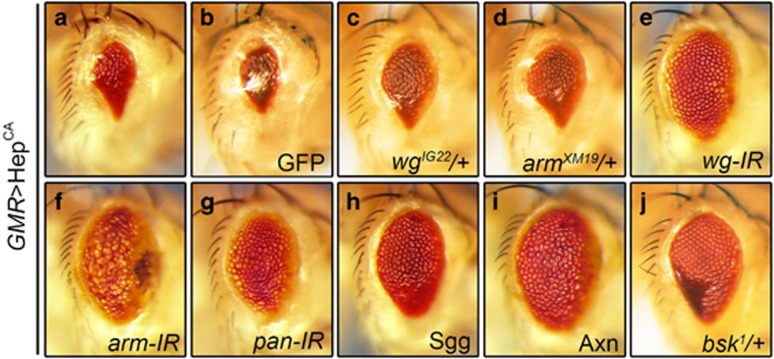
Loss of Wg signaling suppresses Hep-induced Bsk-mediated small eye phenotype. Light micrographs of *Drosophila* adult eyes are shown. Expression of Hep^CA^ triggers cell death and produces a small eye phenotype (**a**), which remains unaffected by expression of GFP (**b**), but is suppressed by mutations in *wg* (**c**) or *arm* (**d**), or RNAi knocking-down of *wg* (**e**), *arm* (**f**) or *pan* (**g**), or expression of Sgg **(h**) or Axn (**i**), while mutation in *bsk* serves as a positive control (**j**)

Egr triggers two independent cell death pathways in *Drosophila*, one mediated by Bsk and another by caspases.^32^ To determine whether Wg pathway is required for Bsk-mediated cell death, we expressed a constitutive active form of Hep (Hep^CA^) encoding the *Drosophila* JNK kinase. Expression of Hep^CA^ in the eye (*GMR*>Hep^CA^) induces Bsk-dependent cell death and generates a small eye phenotype (Figure 2a).^32^ This phenotype is suppressed in heterozygous mutants for *wg* or *arm* (Figures 2c and d), and by knocking-down of *wg*, *arm,* or *pan* (Figures 2e–g) or expression of the negative regulator Sgg or Axn (Figures 2h and i), but remains unaffected by the expression of GFP (Figure 2b). As a positive control, this phenotype is suppressed in heterozygous *bsk* mutants (Figure 2j). Together, these data suggest that Wg signaling is required for Hep-induced small eye phenotype.

### Loss of Wg signaling suppresses Bsk-mediated cell death in other tissues

**Figure 3:**
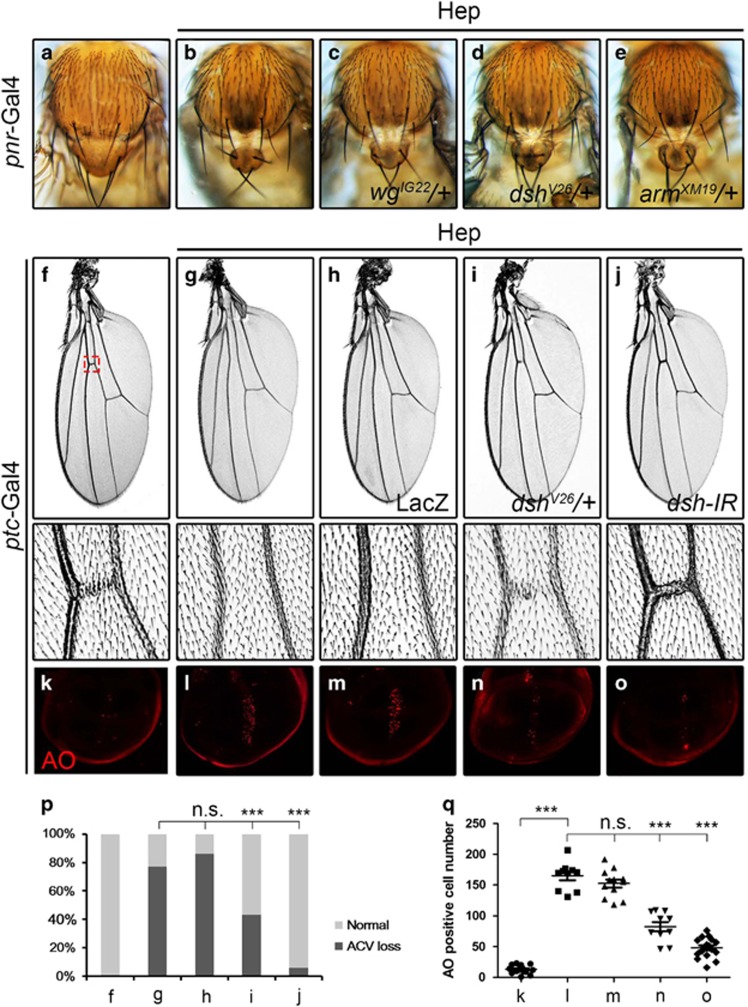
Loss of Wg signaling suppresses Bsk-mediated cell death in other tissues. Light micrographs of *Drosophila* adult notum (**a–e**) and wings (**f–j**), fluorescent micrographs of third instar wing discs (**k–o**) are shown. Compared with the *pnr-*Gal4 control (**a**), ectopic expression of Hep produces a small scutellum phenotype (**b**), which is partially suppressed by mutations in *wg* (**c**), *dsh* (**d**), or *arm* (**e**). Compared with the *ptc*-Gal4 control (**f**), expression of Hep produces a loss-of-ACV phenotype (**g**), which remains unaffected by expression of LacZ (**h**), but is suppressed partially by mutation in *dsh* (**i**) and strongly by RNAi knocking-down of *ds*h (**j**). Compared with the *ptc*-Gal4 control (**k**), expression of Hep induced extensive cell death (**l**), which remains unaffected by expression of LacZ (**m**), but is suppressed significantly by mutation in *dsh* (**n**) and RNAi knocking-down of *dsh* (**o**). (**p**) Statistics of the loss-of-ACV phenotype in (**f**–**j**). (**f**, 0.00%, *n*=203; **g**, 77.23%, *n*=101; **h**, 86.05%, *n*=86; **i**, 43.40%, *n*=53; **j**, 6.08%, *n*=148). Three asterisks, *P*<0.001; n.s., *P*>0.05. (**q**) Statistics of the AO-positive cell number in (**k**–**o**). For each genotype, at least 10 discs were analyzed. Three asterisks, *P*<0.001; n.s., *P*>0.05

To investigate whether Wg signaling modulates Bsk-mediated cell death in other tissues, we activated Bsk signaling in the dorsal thorax or wing. Expression of Hep driven by *pnr*-Gal4 (*pnr*>Hep) triggers Bsk-dependent cell death in the thorax and produces a small scutellum phenotype (Figure 3b).^31^ This phenotype is partially suppressed by mutation in *wg, dsh,* or *arm* (Figures 3c–e). Since knockdown of Wg signaling components in the thorax results in developmental defects (data not shown), their abilities to suppress *pnr*>Hep-induced scutellum phenotype could not be examined. Furthermore, expression of Hep along the anterior/posterior (A/P) compartment boundary driven by *ptc*-Gal4 results in elevated cell death in third instar wing discs as revealed by acridine orange (AO) staining (Figure 3l), and produces loss of the anterior cross vein (ACV) phenotype (Figure 3g). This phenotype is suppressed partially by a mutation in *dsh*, and near fully by the expression of a *dsh* RNAi, but not that of LacZ (Figures 3h–j and p). Consistently, Hep or Egr induced cell death was significantly suppressed by mutation or RNAi-mediated depletion of Wg pathway components (Figures 3m–o and q and Supplementary Figure S3). Collectively, these data suggest that the canonical Wg pathway modulates Bsk-mediated cell death in a non-tissue specific manner.

Compared with the loss-of-ACV phenotype produced by *ptc>*Hep (Figure 3g), *ptc>*Egr was reported to generate a much severe wing phenotype,^32^ presumably due to their different abilities to activate Bsk-mediated cell death. Indeed, Egr induced stronger cell death (AO staining) and Bsk activation (*puc*-LacZ expression) than Hep in the wing disc (Supplementary Figures S3b, e, i and S4).

### Wg signaling promotes caspase-independent cell death

**Figure 4:**
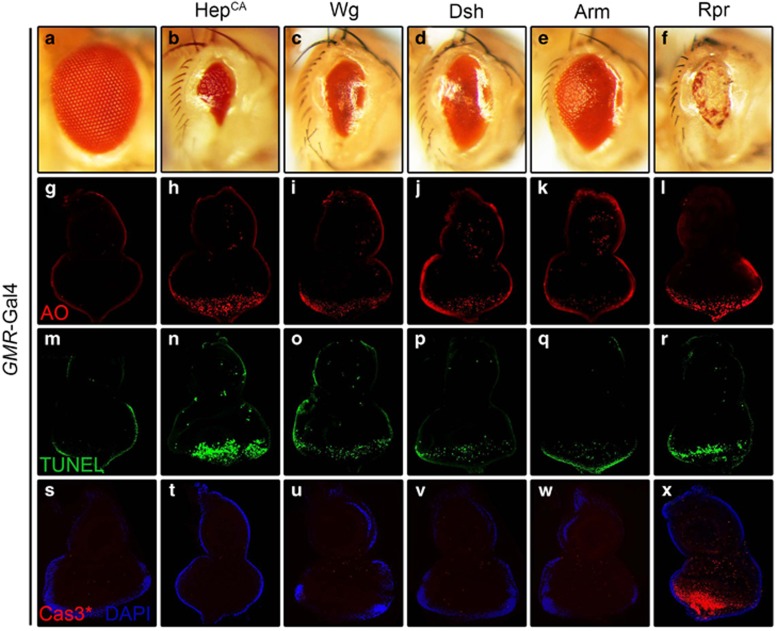
Wg signaling promotes caspase-independent cell death. Light micrographs of *Drosophila* adult eyes (**a–f**) and fluorescent micrographs of third instar eye discs (**g–x**) are shown. Compared with the GMR-Gal4 control (**a**, **g**, **m**, **s**), expression of Hep^CA^, Wg, Dsh, or Arm induces reduced-eye size phenotypes (**b–e**), increased cell death by AO (**h–k**) and TUNEL staining (**n–q**), but no caspase activation indicated by cleaved caspase-3(Cas3*) antibody staining (**t–w**). Expression of Rpr serves as a positive control which induces caspase-dependent cell death (**f**, **l**, **r**, **x**)

To further explore the role of Wg signaling in cell death, we activated the canonical Wg pathway in the developing eye. Expression of Wg, Dsh, or Arm driven by *GMR*-Gal4 promotes extensive cell death as revealed by AO staining (Figures 4i–k) and TUNEL assay (Figures 4o–q) that visualizes dying cells,^38, 39^ and produces eyes with reduced size (Figures 4c–e). All these phenotypes recapitulate that of Bsk activation (Figures 4b, h and n) or expression of the proapoptotic gene Rpr (Figures 4f, l and r). Due to different expression level of each UAS line and the nature of the proteins, the resulted small eye phenotypes exhibit some variation in the size. Consistent with previous study that activated Bsk signaling triggers caspase-independent cell death (Supplementary Figure S5a),^32^ activation of Bsk or Wg signaling does not elicit caspase activation, as detected by an antibody that specifically recognizes the cleaved caspase-3 (Cas3*) (Figures 4s–w). As a positive control, expression of Rpr results in a very strong Cas3* staining (Figure 4x). Furthermore, gain of Wg signaling-triggered small eye phenotypes (Supplementary Figures S5b–d) are not suppressed by deficiency *Df(3 L)H99* (Supplementary Figures S5e–g) that deletes all three proapoptotic genes (*rpr, hid, and grim*), expression of the caspase inhibitor Diap1 (Supplementary Figures S5h–j), a dominant-negative form or RNAi of the initiator caspapse *dronc* (Supplementary Figures S5k–p), or knockdown of the effector caspase *drice* and *dcp-1* (Supplementary Figures S5q–v). Intriguingly, expression of the caspase inhibitor P35 mildly suppressed Wg signaling-triggered small eye phenotypes (Supplementary Figures S6a–c and g–i). Thus, P35 may function as a general cell death inhibitor that blocks both caspase-dependent and -independent cell death. Consistently, expression of P35 also suppresses activated Bsk- or P53-induced cell death phenotype (Supplementary Figures S6d–f and j–l). Besides, Wg signaling-triggered small eye phenotypes are not suppressed by expressing two independent dominant-negative Dp53 (Supplementary Figure S7). Together, these data suggest that activation of Wg signaling contributes to Bsk-mediated, but caspase- and Dp53-independent, cell death *in vivo*.

### Wg signaling is necessary and sufficient for *puc* expression

**Figure 5:**
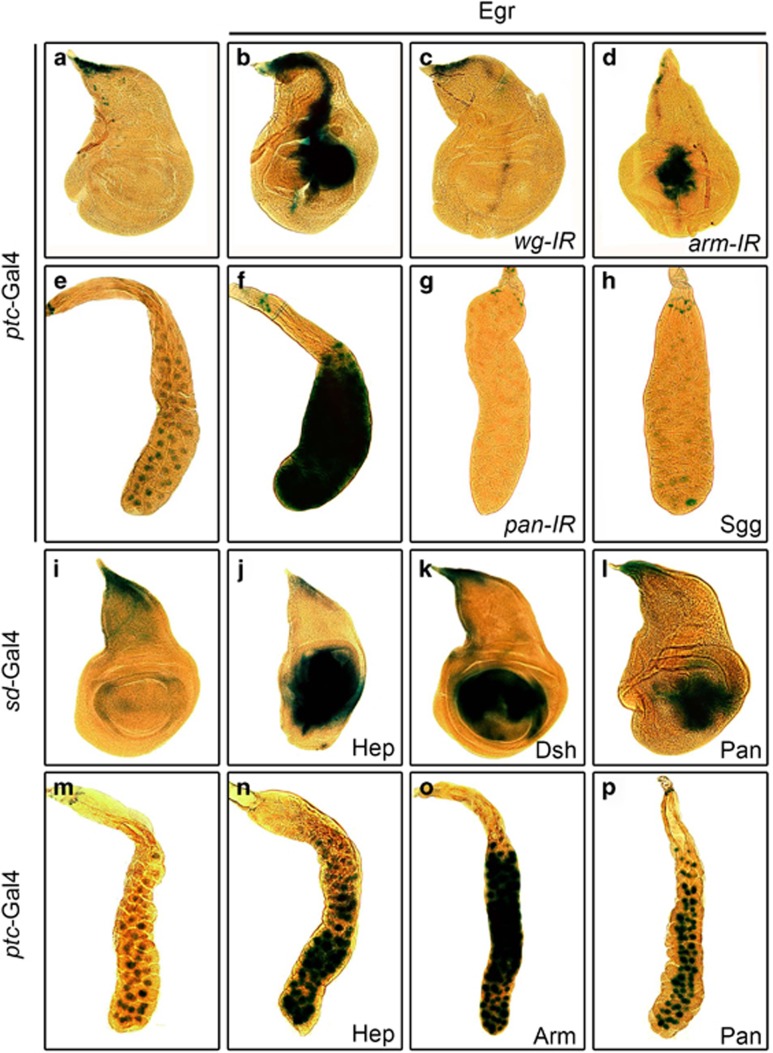
Wg signaling is necessary and sufficient for *puc* expression. Light micrographs of *Drosophila* third instar wing discs (**a–d, i–l**) and salivary glands (**e–h**, **m–p**) with X-Gal staining are shown. Compared with the *ptc-*Gal4 control (**a**, **e**), ectopic Egr-induced *puc-*LacZ expression in wing disc (**b**) or salivary gland (**f**) is suppressed by RNAi knocking-down of *wg* (**c**), *arm* (**d**), or *pan* (**g**), or expression of Sgg (**h**). Compared with the *sd-*Gal4 (**i**) and *ptc-*Gal4 (**m**) control, ectopic expression of Hep (**j**, **n**), Dsh (**k**), Pan (**l**, **p**), or Arm (**o**) triggers *puc-*LacZ expression in wing disc (**j**–**l**) or salivary gland (**n**–**p**)

Activation of Bsk results in transcriptional upregulation of *puc*, which serves as an *in vivo* readout of Bsk signaling.^40^ To investigate whether Wg signaling contributes to Bsk-induced *puc* transcription, we checked the expression of *puc*-LacZ, a LacZ bearing P-element inserted into the *puc* locus (also known as *puc*^*E69*^).^41^ In wild-type wing discs of third instar larva, *puc* is only expressed in the dorsal tip (Figure 5a). Activation of Bsk signaling along the A/P compartment boundary by *ptc>*Egr or *ptc>*Hep induces *puc* expression (Figure 5b and Supplementary Figure S8b), which is significantly suppressed by RNAi-mediated downregulation of canonical Wg signaling (Figures 5c and d, and Supplementary Figures S8c and d). Additionally, activated Bsk also induces *puc* expression in the salivary gland, which is suppressed by blocking Wg signaling (Figures 5e–h).

To examine whether Wg signaling is sufficient to elicit *puc* expression, we activated the canonical Wg pathway in the wing pouch or salivary gland, driven by *sd*-Gal4 or *ptc*-Gal4 respectively. Expression of Hep was included as a positive control. We observed that *puc*-LacZ expression was dramatically enhanced upon activation of Wg or Bsk signaling (Figures 5i–p). Consistently, the expression of endogenous *puc* gene is also elevated by activated Wg or Bsk signaling (Supplementary Figure S8j). However, unlike activation of Bsk signaling, activated Wg pathway cannot activate *puc* expression in the eye discs (Supplementary Figures S8g–i), suggesting that Wg signaling induces *puc* expression in a context-dependent manner. It is likely that additional factor(s) presented only in the wing disc and salivary gland, but not in the eye disc, is required for Wg signaling to induce *puc* expression.

Together, the above data provide compelling evidence that the canonical Wg pathway is both necessary and sufficient for Bsk-induced *puc* expression in both the wing disc and the salivary gland.

### Activation of Bsk signaling promotes *wg* transcription

**Figure 6:**
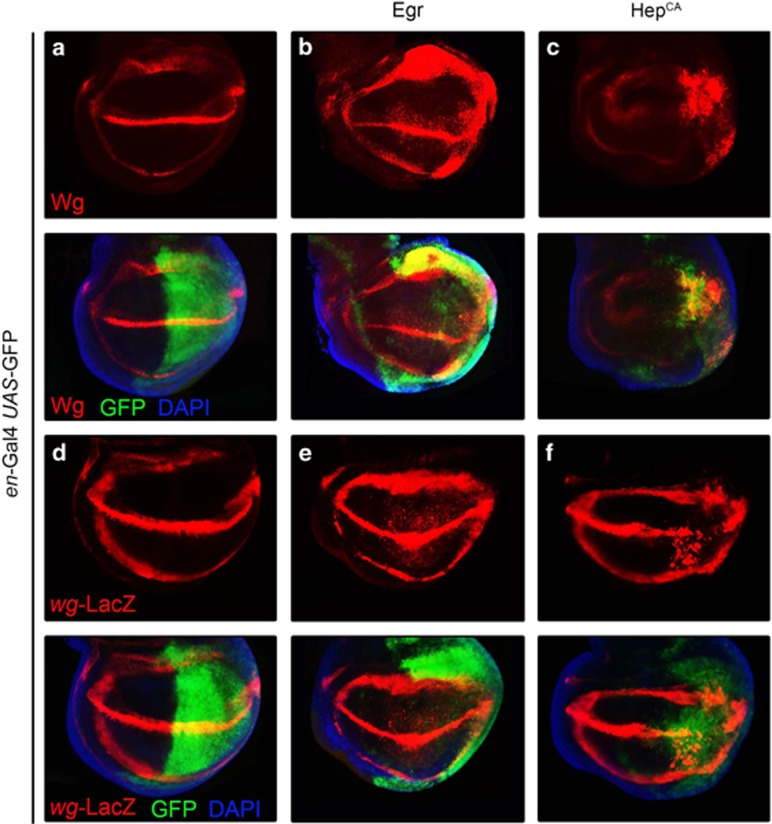
Activation of Bsk signaling promotes *wg* transcription. Fluorescent micrographs of third instar wing pouches are shown. Compared with the *en*-Gal4 UAS-GFP controls (**a**, **d**), Wg (**a**) or *wg*-LacZ (**d**) expression is significantly elevated by ectopic expression of Egr (**b**, **e**) or Hep^CA^ (**c**, **f**) in the posterior compartment (indicated by GFP). For (**c**) and (**f**), flies were reared at 18 °C till early third instar larvae, Hep^CA^ expression was induced at 29 °C for 21 h before dissecting, Wg and *wg*-LacZ expression is detected by anti-Wg and anti-*β*-gal antibody

We have shown that Bsk-mediated cell death requires Wg pathway, which places the Wg signaling downstream of Bsk. Consistent with this explanation, gain of Wg signaling-induced cell death is not suppressed by Bsk^DN^ (Supplementary Figure S9). Given the fact that mutation or RNAi-mediated downregulation of *wg* gene abrogates Bsk-mediated cell death (Figures 1d, e, and i and Supplementary Figure S1b), *wg* expression is probably upregulated by Bsk activation. To test this hypothesis, we first checked Wg protein expression by immunostaining with an anti-Wg antibody. In third instar larva wing discs, endogenous Wg is expressed along the Dorsal/Ventral (D/V) boundary and in two concentric rings in the wing pouch (Figure 6a).^16^ Overexpression of Egr or Hep^CA^ in the posterior compartment of wing disc results in elevated Wg protein level, as compared with that in the anterior compartment (Figures 6b and c). The increased Wg protein level is due to upregulated *wg* transcription, as revealed by a *wg*-LacZ reporter (Figures 6d–f). We also noticed that expression of Hep^CA^ triggers strong cell death (data not shown) that resulted in a dramatic reduction of the posterior compartment (Figures 6c and f). Thus, we conclude that activation of Bsk signaling is able to promote *wg* transcription.

### Wg signaling is required for the physiological functions of Bsk

**Figure 7:**
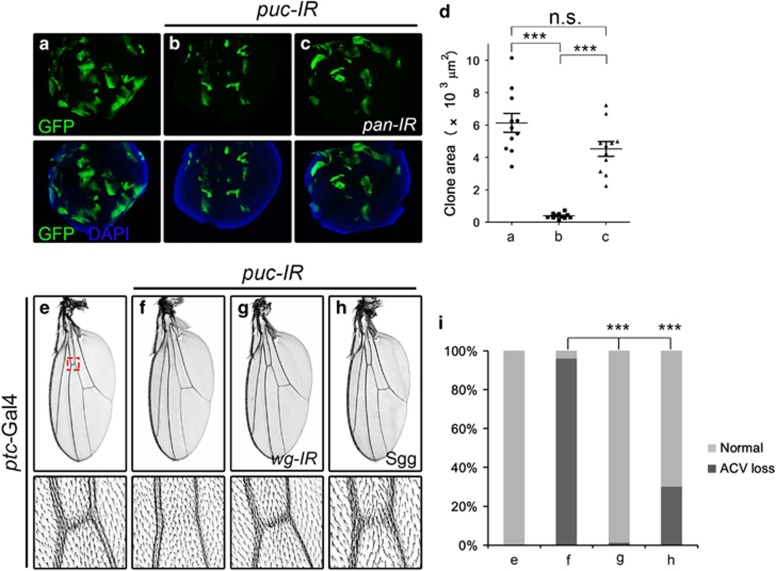
Wg signaling is required for the physiological functions of Bsk. Compared with wild-type clones in the wing pouch (**a**), *puc* loss-of-function clones show dramatically reduced size (**b**), which is fully rescued by RNAi knocking-down of *pan* (**c**). (**d**) Statistics of total clone areas in (**a**–**c**). For each genotype, at least 10 clones were analyzed. Three asterisks, *P*<0.001; n.s., *P*>0.05. Compared with the *ptc*-Gal4 control (**e**), knocking-down of *puc* along the A/P boundary results in a loss-of-ACV phenotype (**f**), which is significantly suppressed by knocking-down of *wg* (**g**) or expression of Sgg (**h**). (**i**) Statistics of the loss-of-ACV phenotype in (**e**–**h**) (**e**, 0.00%, *n*=301; **f**, 96.03%, *n*=126; **g**, 1.25%, *n*=240; **h**, 30.00%, *n*=100). Three asterisks, *P*<0.001

All the above data demonstrate that Wg signaling is required for ectopically activated Bsk-induced cell death, however, it remains unknown whether Wg pathway also contributes to the physiological function of Bsk signaling. To address this question, we generated *puc* loss-of-function clones in *Drosophila* imaginal discs. Previous study suggests that Puc, the Bsk phosphatase, ensures cell viability by restraining basal Bsk signaling, whereas *puc* mutant clones are frequently eliminated from imaginal discs due to Bsk-mediated cell death.^42^ Consistent with this notion, we observed that *puc* loss-of-function clones in wing pouch were considerably smaller than wild-type controls, and could be rescued significantly by blocking Wg signaling (Figures 7a–d). Similar results were also obtained in eye discs (Supplementary Figure S10). Thus, the canonical Wg pathway is critically required for the physiological function of Bsk signaling in regulating epithelia cell viability.

Knocking-down of *puc* along the A/P compartment boundary in developing wings by the *ptc*-Gal4 driver results in a loss-of-ACV phenotype (Figure 7f). This phenotype resembles that of Bsk activation (Figure 3g), and is significantly rescued by downregulation of Wg pathway (Figures 7g–i), suggesting that Wg pathway contributes to the physiological function of Bsk signaling in vein development.

Loss of cell polarity genes, including s*cribble (scrib)*, *lethal giant larvae (lgl)* and *disc large (dlg),*^43^ results in Bsk-mediated cell death and elimination from the tissue.^44, 45^ Knocking-down of *lgl* along the A/P compartment boundary (*ptc*>*lgl-IR*) in the wing pouch induces Bsk-dependent cell death (Supplementary Figures S11a–d and g), which is significantly suppressed by depletion of *wg* or *dsh* (Supplementary Figures S11e–g), suggesting that the Wg pathway is also required for loss-of-cell-polarity induced Bsk-mediated cell death.

## Discussion

The interplay between JNK signaling and the non-canonical Wnt/Fz-PCP pathway has been extensively studied, in which JNK acts downstream of Dsh through Rho family small GTPase, and regulates the key effectors such as Myosin II and other cell adhesion components.^26, 27, 28^ PCP establishment has vital roles in the pattern formation of ommatidia, wing hair and thoracic bristles in *Drosophila,*^27^ and in axis elongation, neural tube closure, inner ear pattering and wound healing in vertebrate.^46, 47^ On the other hand, relative little is known about the crosstalk between JNK and the canonical Wnt/Wg pathways. Previous study has suggested a collaboration between Bsk and the canonical Wg pathway during *Drosophila* embryogenesis. However, the two signaling routes appear to function in parallel, and the underlying mechanism remains unclear.^29^ Apoptotic cells, when kept alive ('undead cells') by expressing P35, induce compensatory proliferation in neighbor cells by secreting growth factors like Wg, Dpp and Hh.^48, 49, 50^ Both secretion of growth factors and compensatory proliferation depends on Bsk signaling,^4, 51, 52^ yet the mechanism has not been fully illustrated. In mammal, a connection between c-Jun and TCF4, transcription factors of JNK and Wnt pathways respectively, is reported to have a role in intestinal tumorigenesis.^30^

In the present study, we characterize the genetic interaction between Bsk and the canonical Wg signaling in *Drosophila*, and obtain the following results: (1) loss of Wg signaling suppresses Bsk-mediated caspase-independent cell death and *puc* expression; (2) activation of Wg signaling induces caspase-independent cell death and *puc* expression, which challenges the previous opinion that *puc* is a direct transcription target and readout of Bsk signaling. We cannot rule out the possibility that a context-dependent feed-back loop may exist between Wg and Bsk signaling; (3) Wg signaling acts downstream of Bsk in promoting cell death; (4) Wg pathway participates in the physiological function of Bsk signaling; (5) activated Bsk signaling results in upregulated *wg* transcription. Thus, we not only deliver compelling evidences for the conclusion that activated Bsk signaling promotes Wg pathway-dependent cell death, but also provide the underlying mechanism for the interplay of the two pathways that have crucial roles in development. However, since Wg signaling is necessary for cell proliferation and viability, complete loss of Wg signaling would result in developmental defect and animal lethality, we used heterozygous mutants or RNAi-mediated knock-down approach to examine the effect of loss-of-Wg signaling on Bsk-mediated cell death. In such backgrounds, Wg signaling is effectively reduced, but not completely abolished. Consequently, Bsk-mediated cell death and resulted phenotypes are significantly, but not fully, suppressed (Figures 1, 2, 3). Therefore, we could not exclude the possibility that other factor(s) or signaling pathway(s) may exist in parallel with Wg signaling to mediate Bsk signaling-induced cell death (Supplementary Figure S5a).

As an evolutionary conserved signaling pathway, *Drosophila* Wg signaling has been shown to have important roles for developmentally regulated cell death, like sculpting the retina and ommatidia.^53, 54, 55^ Previous works suggested that Wg signaling induces ommatidial elimination through elevated expression of pro-apoptotic factors *rpr, hid* and *grim.*^54^ In contrast with the finding, we show Wg signaling induces caspase-independent cell death, evident by the fact that no cleaved caspase 3 is detected by antibody staining (Figures 4u–w), and Wg signaling-triggered cell death are not suppressed by *Df(3L)H99* and expression of Diap1, a dominant-negative form or knockdown of *dronc*, or knockdown of *drice* and *dcp-1* (Supplementary Figures S5b–v). One plausible explanation for this discrepancy is that Wg signaling may promote cell death via distinct mechanisms in a context-dependent manner. Since Bsk is also involved in necroptosis triggered by factors such as the mitochondrial protein apoptosis-inducing factor (AIF),^56, 57^ it would be interesting to investigate in future whether Wg signaling-induced caspase-independent cell death has a role in necroptosis.

Since the discovery of the first *Wnt* gene more than 30 years ago,^58^ research on Wnt signaling has growing into one of the most active field-, and huge amount of progress have been achieved at an accelerating pace.^59^ Given the numerous functions of Wnt signaling during development, it is not surprising that deregulation of this pathway is a prevalent theme in cancer biology, especially colorectal cancer (CRC). Constitutively active Wnt signaling has been associated with tumor progression in many cancers, yet in this study we characterize a role of Wg signaling in promoting cell death, which implies that Wnt signaling may also possess a tumor suppressor function. Meanwhile, a substantial body of evidence also suggests that JNK signaling is closely related with tumor formation and metastasis,^60, 61, 62, 63, 64^ thus the present study shed new light on the crosstalk and involvement of JNK and Wnt signaling in cell death and cancer development.

## Materials and Methods

### *Drosophila* strains and generation of clones

The following stocks have been described previously: *wg*^1–17^;^65^ *Sp*;^34^ *dsh*^6^;^66^ *wg*^*IG22*^, *dsh*^*V26*^, *arm*^XM19^;^67^ *arm*^1^, *pan*^*13a*^;^68^ Sgg^EP1576^;^69^ *UAS-*Wg, *UAS-*Dsh, *UAS-wg-IR* and *UAS-arm-IR;*^70^ *UAS-*Pan;^71^ *bsk*^1^; ^72^ *UAS-*Egr^Regg1^;^10^ *UAS-*Hep^CA^, *UAS-*Egr, *UAS-*Bsk^DN^, *UAS-*Puc, *Df(3 L)H99*, DIAP1, DRONC^DN^ and *GUS-*Dp53^259H^;^32^ *UAS-*Hep^WT^;^31^ *puc*^*E69*^, *pnr-*Gal4;^12^ *GMR-*Gal4, *ptc-*Gal4 and *sd-*Gal4;^73^ *UAS-*Dp53^H159N^.^35^ The *wg*-LacZ, *UAS-*Rpr, *UAS-*Dp53, *UAS-*Arm, *UAS-*Apc2, *UAS-*Axn, and *UAS-dsh-IR* lines were obtained from the Bloomington stock center. The *UAS-pan-IR* and *UAS-lgl-IR* lines were obtained from the Vienna Drosophila RNAi Center (VDRC). The *UAS-wg-IR*, *UAS-arm-IR, UAS-dronc-IR, UAS-drice-IR, UAS-dcp-1-IR* lines were got from Fly Stocks of National Institute of Genetics (NIG-FLY).

Fluorescently labelled clones were produced in larval imaginal discs using the *y w*^*1118*^ *hs*-Flp; *act*>CD2>Gal4 *UAS*-GFP/Cyo strain. Clones were induced by heat shock at 37 °C for 10 min, late third-instar larvae were dissected after recovering for 3 days.

### Immunostaining

The following primary antibodies were used: 1 : 400 rabbit-anti-Caspase 3 (Cell Signaling Technology, CST, Cat # 9661, Danvers, MA, USA), 1 : 300 mouse-anti-Wingless (Developmental Studies Hybridoma Bank, DSHB, lova City, IA, USA), 1 : 500 mouse-anti-*β*-Gal (DSHB). The second antibodies were used as follows: 1 : 1000 anti-mouse CY3 (CST), 1 : 1000 anti- rabbit CY3 (CST).

### AO staining

The discs were dissected at the third instar larva stage, and stained for AO as described.^32^

### TUNEL staining

The discs were dissected at the third instar larva stage, and stained for TUNEL using the Fluorescein Cell Death Kit produced by Boster Company. Imaging of prepared sample was conducted by a Leica confocal microscope (Leica SP5, Solms, Germany).

### X-Gal staining

The discs were dissected at the third instar larva stage, and stained for *β*-galactosidase as described.^74^

### qRT-PCR

Wing discs were dissected at third instar larva, for each genotype, more than 200 discs were collected and total RNA was isolated using TRIzol (Invitrogen, Carlsbad, CA, USA). qRT-PCR was performed as previously described,^75^ primers pairs of *rp49*^75^ and *puc*^76^ were used as previously described.

### Statistics

For loss of ACV phenotype, statistics were analyzed using chi-square test. For AO stanining and area of disc clones, one-way analysis of variance test followed by the post Dunnett test or Kruskal–Wallis text followed by the post Dunns text was used. A *P*-value of <0.05 was considered as significant.

## Abbreviations

JNK: c-Jun N-terminal kinase
TNF: tumor necrosis factor
APC: adenomatous polyposis coli protein
CRC: colorectal cancer
PCP: planar cell polarity
GMR: glass multiple reporter
ACV: anterior cross vein
A/P: anterior/posterior
D/V: dorsal/ventral
AO: acridine orange
RNAi: RNA interference

## Supplementary Material

Supplemetary Information
