# Supplementary material for: The canonical Wg signaling modulates Bsk-mediated cell death in Drosophila
Source: Cell Death Dis. 2015 Apr 9;6(4):e1713–. doi: 10.1038/cddis.2015.85 (PMC4650552; doi:10.1038/cddis.2015.85)
Supplement: Supplemetary Information [file cddis201585x1.pdf]

Supplementary Information

Zhang et al., Figure S1

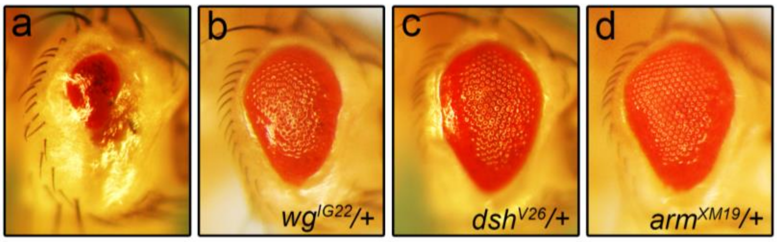

Figure S1. Loss of Wg signaling suppresses Egr-induced cell death in eyes

Light micrographs of *Drosophila* adult eyes are shown. *GMR*>Egr induced small eye phenotype **(a)** is strongly suppressed by mutations in *wg* **(b)**, *dsh* **(s)** or *arm* **(d)**.

Zhang et al., Figure S2

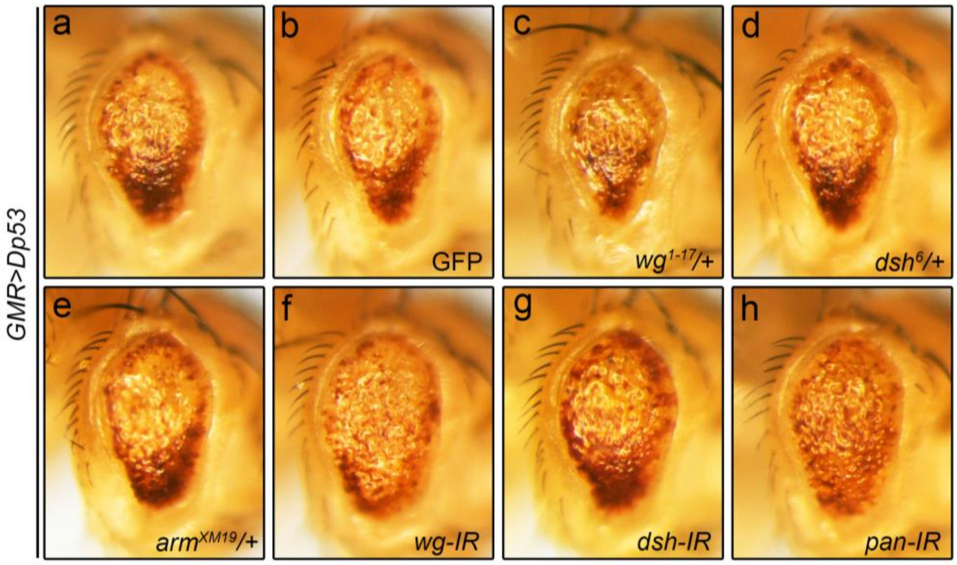

Figure S2. Loss of Wg signaling does not suppress Dp53-induced cell death phenotype

Expression of Dp53 under *GMR*-Gal4 generates a small and rough eye phenotype (**a**), which remains unaffected by expression of GFP (**b**), mutations in *wg* (**c**), *dsh* (**d**) or *arm* (**e**), and RNAi-mediated knocking-down of *wg* (**f**), *dsh* (**g**) or *pan* (**h**).

Zhang et al., Figure S3

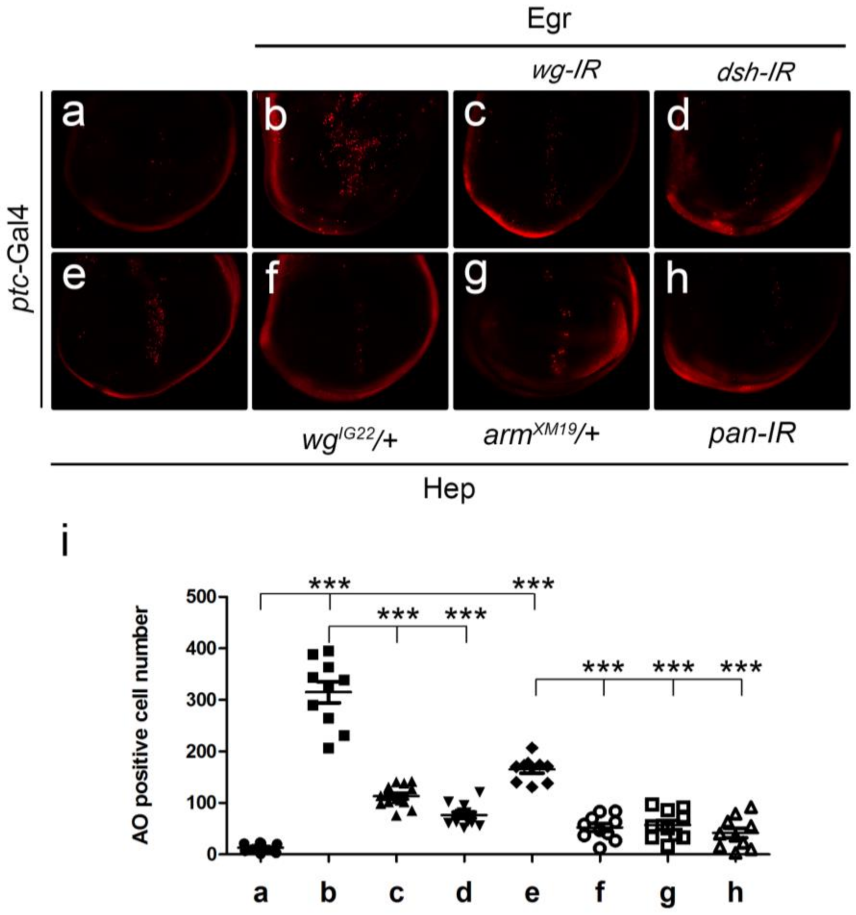

Figure S3. Loss of Wg signaling suppress Bsk-mediated cell death in the wing discs

Fluorescent micrographs of 3rd instar wing discs **(a-h)** are shown. Compared with the *ptc*-Gal4 control **(a)**, expression of Egr induces extensive cell death **(b),** which is suppressed significantly by RNAi knocking-down of *wg* (**c**) or *dsh* (**d**). Expression of Hep induces weaker cell death **(e)**, which is also suppressed significantly by mutation in *wg* (**f**) or *arm* (**g**) and RNAi knocking-down of *pan* **(h)**. **(i)** Statistics of the AO positive cell number in a-h. For each genotype, at least 10 discs were analyzed. Three asterisks, $p < 0.001$.

Zhang et al., Figure S4

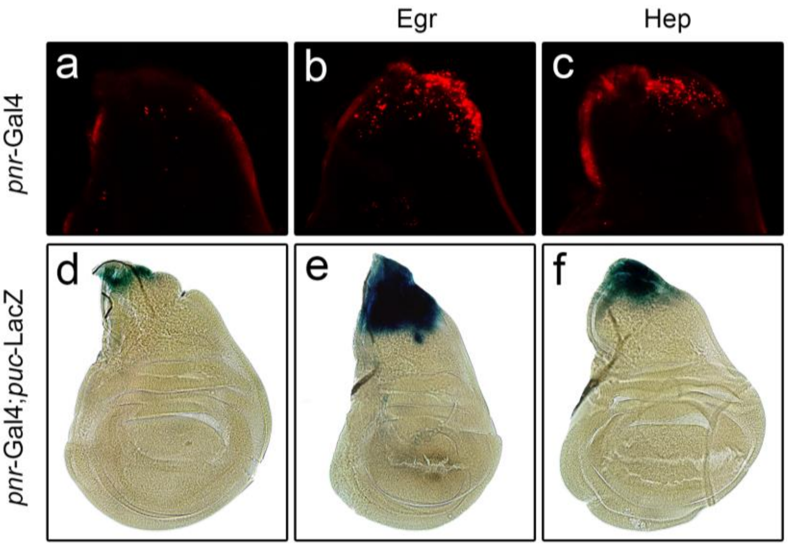

Figure S4. Egr induces more Bsk-activation and cell death than Hep

Fluorescent **(a-c)** and light **(d-f)** micrographs of 3rd instar wing discs are shown. Compared with the *pnr*-Gal4 control **(a, d)**, expression of Egr induces much stronger cell death **(b**, AO staining**)** and *puc*-LacZ expression **(**e, X-Gal staining**)** than that of Hep **(c**, **f)**.

Zhang et al., Figure S5

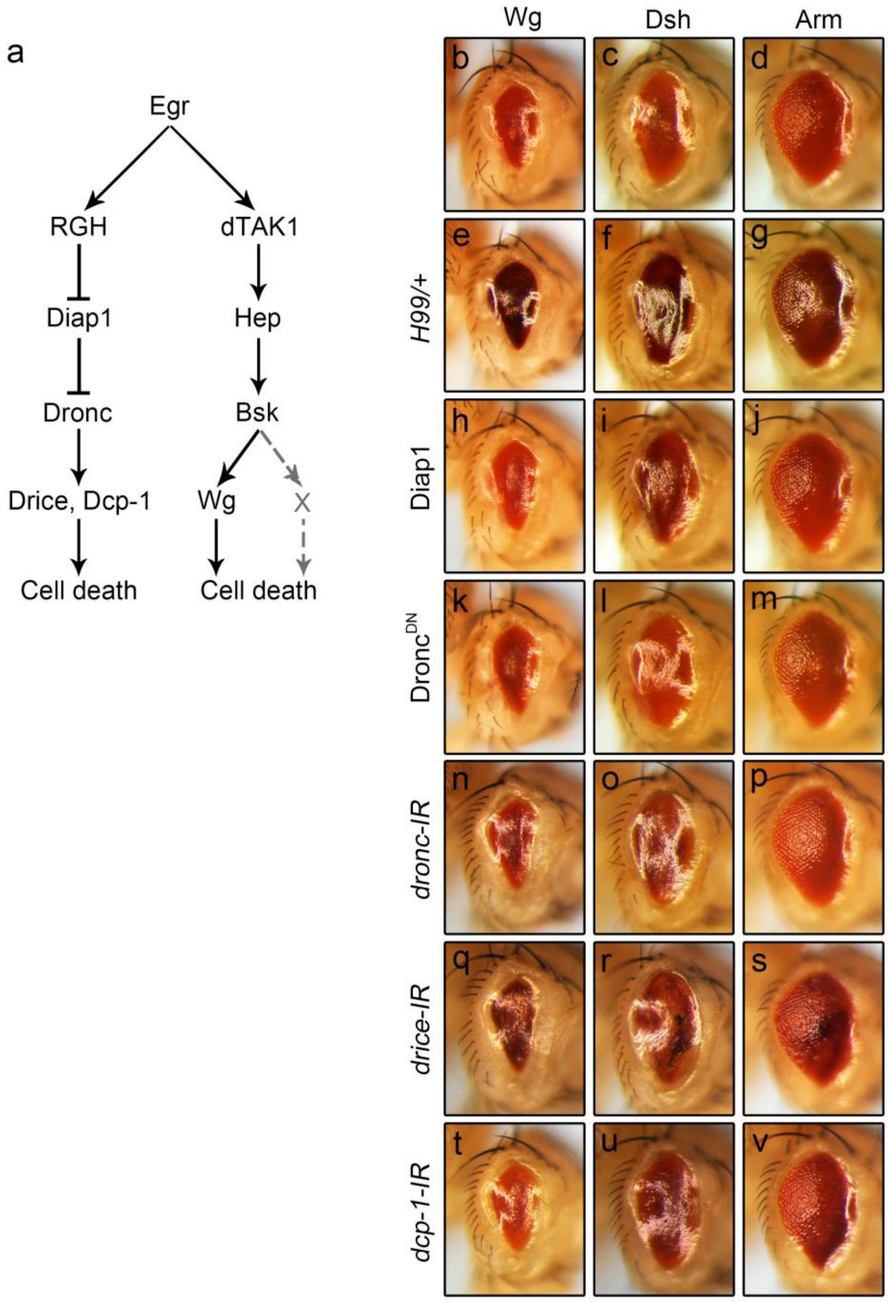

Figure S5. Wg signaling promotes caspase-independent cell death

(a) A model showing Egr-induced cell death is mediated by two distinct pathways. **(b-v)** Light micrographs of *Drosophila* adult eyes are shown. The small eye phenotype triggered by ectopic expression of Wg (**b**), Dsh (**c**) or Arm (**d**) cannot be suppressed by deficiency *Df(3L)H99* (**e-g**), expression of Diap1 (**h-j**), a dominant negative form of DRONC (**k-m**), and RNAi knocking-down of *dronc* (**n-p**), *drice* (**q-s**) or *dcp-1*(**t-v**).

Zhang et al., Figure S6

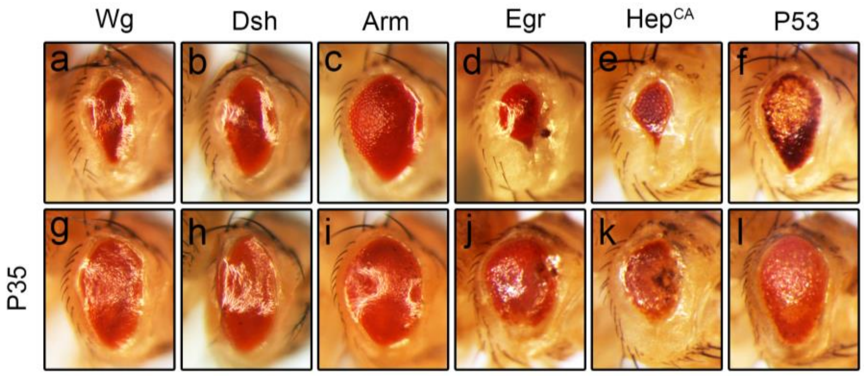

Figure S6. P35 is a general suppresser of cell death

Light micrographs of *Drosophila* adult eyes are shown. The small eye phenotype induced by ectopic Wg, Dsh, Arm, Egr, Hep^{CA} or P53 **(a-f)** is suppressed by P35 **(g-l)**.

Zhang et al., Figure S7

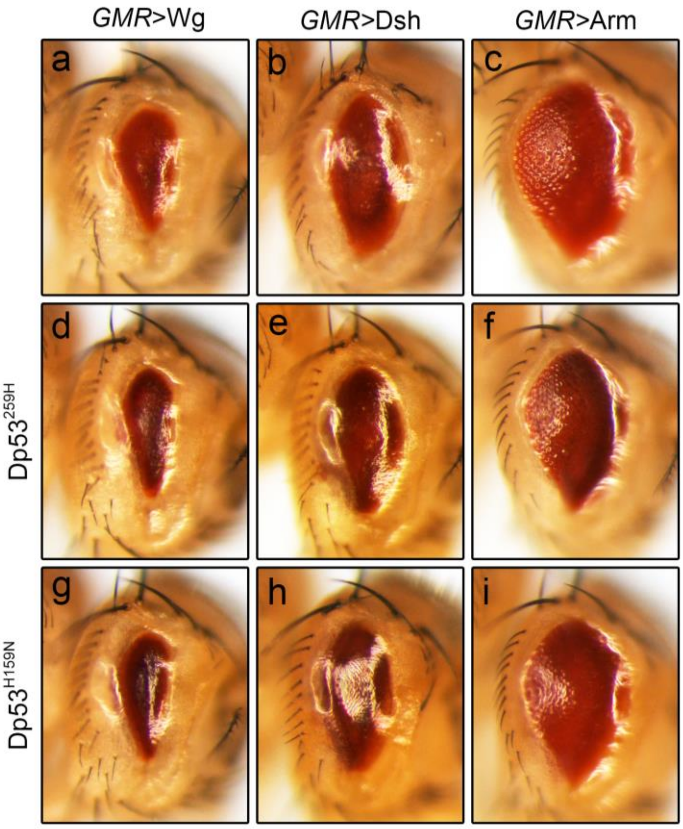

Figure S7. Wg signaling-induced cell death is independent of Dp53

Light micrographs of *Drosophila* adult eyes are shown. Ectopic Wg, Dsh or Arm induced small eye phenotype **(a-c)** cannot be suppressed by the expression of two independent dominant negative forms of Dp53 **(d-i)**.

Zhang et al.,Figure S8

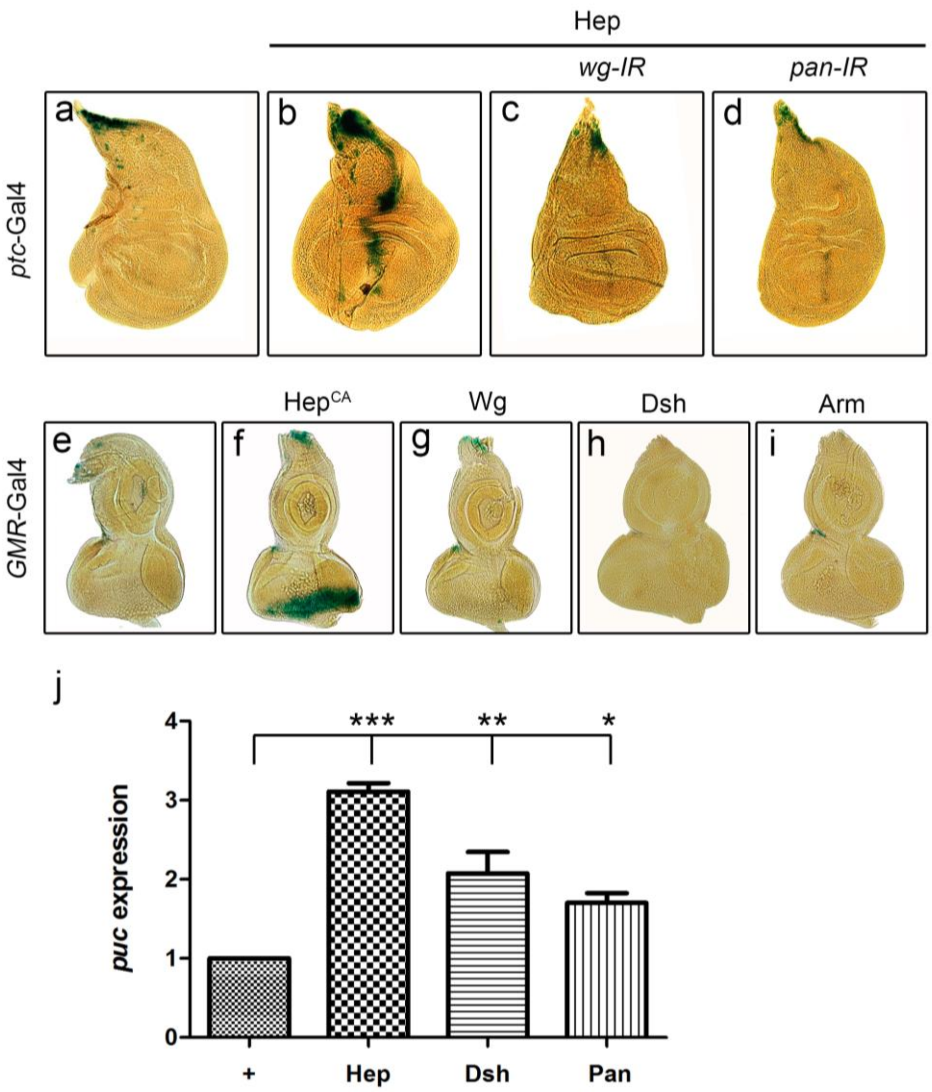

Figure S8. Wg signaling modulates Bsk activation in a tissue specific manner

Light micrographs of *Drosophila* 3rd instar wing **(a-d)** and eye discs **(e-i)** with X-Gal staining are shown. Compared with the *ptc*-Gal4 control **(a)**, ectopic expression of Hep along the A/P compartment boundary in the wing disc activates *puc*-LacZ expression (**b**), which is suppressed by knocking-down *wg* (**c**) or *pan* (**d**). Compared with the *GMR*-Gal4 control **(e)**, ectopic expression of Hep^{CA} **(f)**, but not Wg **(g)**, Dsh **(h)** or Arm **(i)**, activates *puc*-LacZ expression in the eye discs**. (j)** Quantification of *puc* expression level in 3rd instar wing discs by qRT-PCR. Compared with the *sd*-Gal4 control (+), expression of Hep, Dsh, or Pan significantly activates *puc* expression. Three asterisks, $p < 0.001$. Two asterisks, $p < 0.01$. One asterisks, $p < 0.05$.

Zhang et al., Figure S9

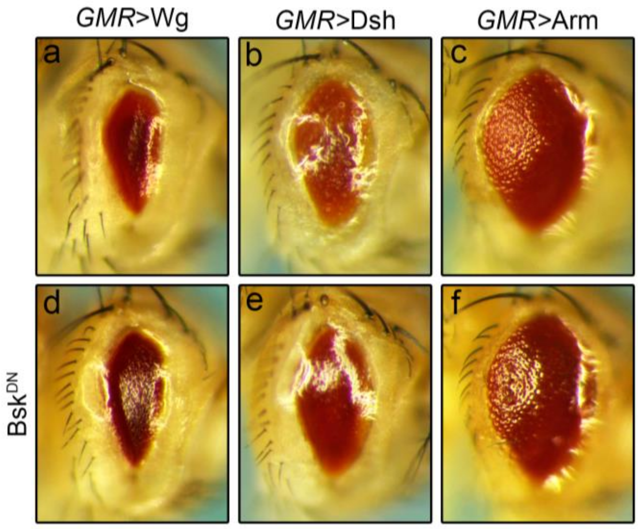

Figure S9. Wg signaling acts downstream of Bsk

Light micrographs of *Drosophila* adult eyes are shown. Ectopic Wg (**a**), Dsh (**b**) or Arm (**c**) induced small eye phenotype cannot be suppressed by expression of Bsk^{DN} **(d-f)**.

Zhang et al., Figure S10

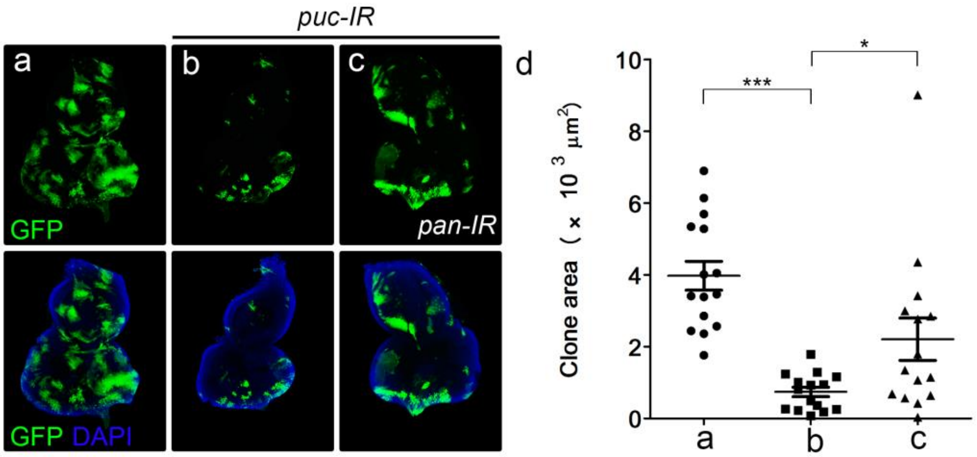

Figure S10. Wg signaling contributes to the physiological function of Bsk

(a-c) Fluorescent micrographs of 3rd instar eye discs are shown. Compared with wild type clones induced in the eye disc **(a)**, *puc* loss-of-function clones show dramatically reduced area **(b)**, which is partially rescued by RNAi knocking-down of *pan* **(c)**. **(d)** Statistics of the total clone areas in a-c. For each genotype, at least 15 clones were analyzed. Three asterisks, $p < 0.001$; one asterisks, $p < 0.05$.

Zhang et al., Figure S11

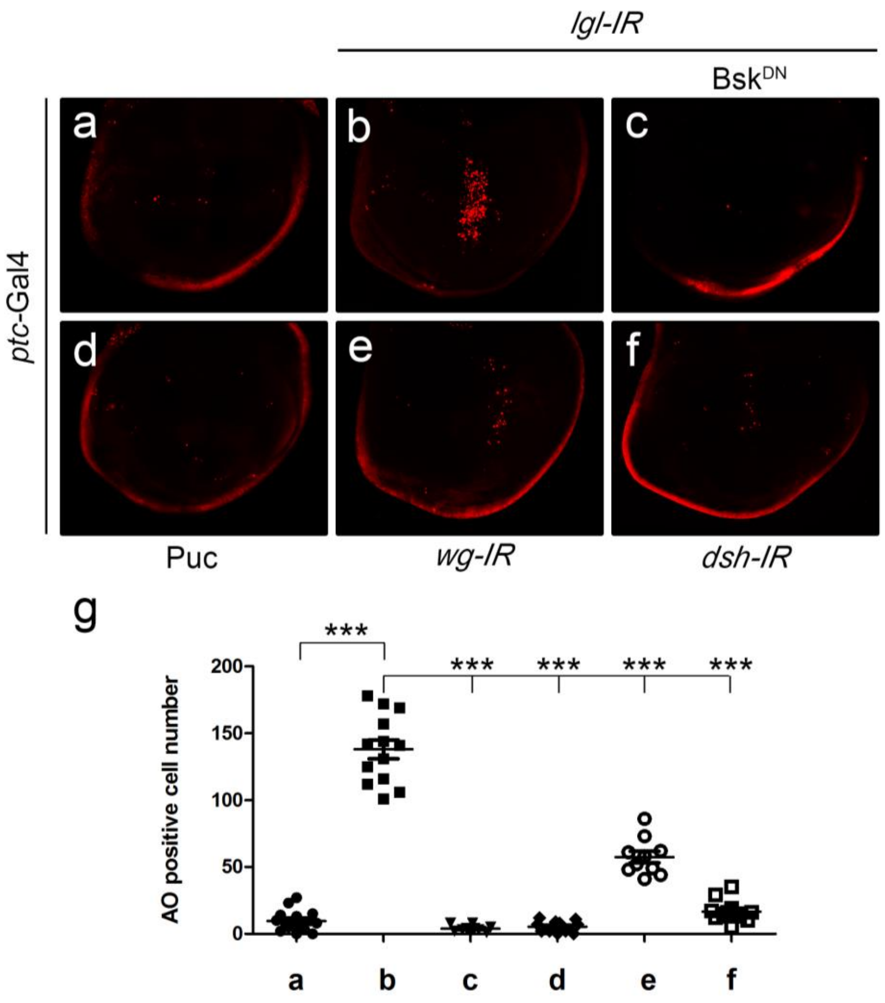

Figure S11. Wg signaling modulates loss of cell polarity-induced Bsk-mediated cell death

(a-f) Fluorescent micrographs of 3rd instar wing discs are shown. Compared with the *ptc*-Gal4 control **(a)**, depletion of *lgl* induces extensive cell death **(b),** which is suppressed by expression of Bsk^{DN} **(c)** or Puc **(d)**, or RNAi-mediated knocking-down of *wg* **(e)** or *dsh* **(f)**. For each genotype, at least 10 discs were analyzed. Three asterisks, $p < 0.001$.

Detailed Genotypes

Figure 1

(a) *GMR*-Gal4/+

(b) *UAS*-Egr^{Regg1}/+; *GMR*-Gal4/+

(c) *UAS*-Egr^{Regg1}/+; *GMR*-Gal4/*UAS*-GFP

(d) *UAS*-Egr^{Regg1}/*Sp*; *GMR*-Gal4/+

(e) *UAS*-Egr^{Regg1}/*wg*$^{1\text{-}17}$; *GMR*-Gal4/+

(f) *dsh*^{6}/+; *UAS*-Egr^{Regg1}/+; *GMR*-Gal4/+

(g) *arm*^{1}/+; *UAS*-Egr^{Regg1}/+; *GMR*-Gal4/+

(h) *UAS*-Egr^{Regg1}/+; *GMR*-Gal4/+; *pan*^{13a}/+

(i) *UAS*-Egr^{Regg1}/+; *GMR*-Gal4/*UAS-wg-IR*

(j) *UAS*-Egr^{Regg1}/+; *GMR*-Gal4/*UAS-dsh-IR*

(k) *UAS*-Egr^{Regg1}/+; *GMR*-Gal4/*UAS-pan-IR*

(l) Sgg^{EP1576}/+; *UAS*-Egr^{Regg1}/+; *GMR*-Gal4/+

(m) *UAS*-Egr^{Regg1}/+; *GMR*-Gal4/*UAS*-Apc2

(n) *UAS*-Egr^{Regg1}/*bsk*^{1}; *GMR*-Gal4/+

(o) *UAS*-Egr^{Regg1}/+; *GMR*-Gal4/*UAS*-Bsk^{DN}

Figure 2

(a) *GMR*-Gal4 *UAS*-Hep^{CA}/+

(b) *GMR*-Gal4 *UAS*-Hep^{CA}/*UAS*-GFP

(c) *wg*^{IG22}/+; *GMR*-Gal4 *UAS*-Hep^{CA}/+

(d) *arm*^{XM19}/+; *GMR*-Gal4 *UAS*-Hep^{CA}/+

(e) *GMR*-Gal4 *UAS*-Hep^{CA} /*UAS-wg-IR*

(f) *GMR*-Gal4 *UAS*-Hep^{CA}/*UAS-arm-IR*

(g) *GMR*-Gal4 *UAS*-Hep^{CA}/*UAS-pan-IR*

(h) Sgg^{EP1576}/+; *GMR*-Gal4 *UAS*-Hep^{CA}/+

(i) *GMR*-Gal4 *UAS*-Hep^{CA}/*UAS*-Axn

(j) *bsk*^{1}/+; *GMR*-Gal4 *UAS*-Hep^{CA}/+

Figure 3

(a) *pnr*-Gal4/+

(b) *UAS*-Hep/+; *pnr*-Gal4/+

(c) *UAS*-Hep/wg^{IG22}; *pnr*-Gal4/+

(d) dsh^{V26}/+; *UAS*-Hep/+; *pnr*-Gal4/+

(e) arm^{XM19}/+; *UAS*-Hep/+; *pnr*-Gal4/+

(f) *ptc*-Gal4/+; puc^{E69}/+

(g) *ptc*-Gal4 *UAS*-Hep/+; puc^{E69}/+

(h) *ptc*-Gal4 *UAS*-Hep/+; puc^{E69}/*UAS*-LacZ

(i) dsh^{V26}/+; *ptc*-Gal4 *UAS*-Hep/+; puc^{E69}/+

(j) *ptc*-Gal4 *UAS*-Hep/+; puc^{E69}/*UAS-dsh-IR*

(k) *ptc*-Gal4/+

(l) *ptc*-Gal4 *UAS*-Hep/+

(m)*ptc*-Gal4 *UAS*-Hep/+; *UAS*-LacZ/+

(n) dsh^{V26}/+; *ptc*-Gal4 *UAS*-Hep/+

(o) *ptc*-Gal4 *UAS*-Hep/+; *UAS-dsh-IR/+*

Figure 4

(a, g, m and s) *GMR*-Gal4/+

(b, h, n and t) *GMR*-Gal4 *UAS*-Hep^{CA}/+

(c, i, o and u) *GMR*-Gal4 *UAS*-Wg/+

(d, j, p and v) *GMR*-Gal4 *UAS*-Dsh/+

(e, k, q and w) *UAS*-Arm/+; *GMR*-Gal4/+

(f, l, r and x) *UAS*-Rpr/+; *GMR*-Gal4/+

Figure 5

(a, e and m) *ptc*-Gal4/+; puc^{E69}/+

(b, f) *ptc*-Gal4 *UAS*-Egr/+; puc^{E69}/+

(c) *ptc*-Gal4 *UAS*-Egr/+; puc^{E69}/*UAS-wg-IR*

(d) *ptc*-Gal4 *UAS*-Egr/+; *puc*^{E69}/*UAS-arm-IR*

(g) *ptc*-Gal4 *UAS*-Egr/+; *puc*^{E69}/*UAS-pan-IR*

(h) Sgg^{EP1576}/+; *ptc*-Gal4 *UAS*-Egr/+; *puc*^{E69}/+

(i) *sd*-Gal4/+; *puc*^{E69}/+

(j) *sd*-Gal4/+; *UAS*-Hep/+; *puc*^{E69}/+

(k) *sd*-Gal4/+; *puc*^{E69}/*UAS*-Dsh

(l) *sd*-Gal4/+; *UAS*-Pan/+; *puc*^{E69}/+

(n) *ptc*-Gal4/*UAS*-Hep; *puc*^{E69}/+

(o) *ptc*-Gal4/*UAS*-Arm; *puc*^{E69}/+

(p) *ptc*-Gal4/*UAS*-Pan; *puc*^{E69}/+

Figure 6

(a) *en*-Gal4 *UAS*-GFP/+

(b) *en*-Gal4 *UAS*-GFP/*UAS*-Egr^{Regg1}

(c) *en*-Gal4 *UAS*-GFP/+; *tub*-Gal80^{ts}/*UAS*-Hep^{CA}

(d) *en*-Gal4 *UAS*-GFP/*wg*-LacZ

(e) *en*-Gal4 *UAS*-GFP/ *wg*-LacZ *UAS*-Egr^{Regg1}

(f) *en*-Gal4 *UAS*-GFP/*wg*-LacZ; *tub*-Gal80^{ts}/*UAS*-Hep^{CA}

Figure 7

(a) *hs*-Flp; *act*>CD2>Gal4 *UAS*-GFP/+

(b) *hs*-Flp; *act*>CD2>Gal4 *UAS*-GFP/*UAS-puc-IR*

(c) *hs*-Flp; *act*>CD2>Gal4 *UAS*-GFP/*UAS-puc-IR; UAS-pan-IR*/+

(e) *ptc*-Gal4/+

(f) *ptc*-Gal4 *UAS-puc-IR*/+

(g) *ptc*-Gal4 *UAS-puc-IR*/+; *UAS-wg-IR/*+

(h) Sgg^{EP1576}/+; *ptc*-Gal4 *UAS-puc-IR*/+

Figure S1

(a) *UAS*-Egr^{Regg1}/+; *GMR*-Gal4/+

(b) *UAS*-Egr$^{\mathrm{Regg1}}$/*wg*^{IG22}; *GMR*-Gal4/+

(c) *dsh*^{V26}/+; *UAS*-Egr$^{\mathrm{Regg1}}$/+; *GMR*-Gal4/+

(d) *arm*^{XM19}/+; *UAS*-Egr$^{\mathrm{Regg1}}$/+; *GMR*-Gal4/+

Figure S2

(a) *GMR*-Gal4 *UAS*-Dp53/+

(b) *GMR*-Gal4 *UAS*-Dp53/+;*UAS*-GFP/+

(c) *GMR*-Gal4 *UAS*-Dp53/*wg*$^{1\text{-}17}$

(d) *dsh*^{6}/+; *GMR*-Gal4 *UAS*-Dp53/+

(e) *arm*^{XM19}/+;*GMR*-Gal4 *UAS*-Dp53/+

(f) *GMR*-Gal4 *UAS*-Dp53/+; *UAS-wg-IR*/+

(g) *GMR*-Gal4 *UAS*-Dp53/+; *UAS-dsh-IR*/+

(h) *GMR*-Gal4 *UAS*-Dp53/+; *UAS-pan-IR*/+

Figure S3

(a) *ptc*-Gal4/+

(b) *ptc*-Gal4 *UAS*-Egr/+

(c) *ptc*-Gal4 *UAS*-Egr/+; *UAS-wg-IR*/+

(d) *ptc*-Gal4 *UAS*-Egr/+; *UAS-dsh-IR*/+

(e) *ptc*-Gal4 *UAS*-Hep/+

(f) *ptc*-Gal4 *UAS*-Hep/*wg*^{IG22}

(g) *arm*^{XM19}/+; *ptc*-Gal4 *UAS*-Hep/+

(h) *ptc*-Gal4 *UAS*-Hep/+; *UAS-pan-IR/+*

Figure S4

(a) *pnr*-Gal4/+

(b) *UAS*-Egr/+; *pnr*-Gal4/+

(c) *UAS*-Hep/+; *pnr*-Gal4/+

(d) *pnr*-Gal4/*puc*^{E69}

(e) *UAS*-Egr/+; *pnr*-Gal4/*puc*^{E69}

(f) *UAS*-Hep/+; *pnr*-Gal4/*puc*^{E69}

Figure S5

(b) *GMR*-Gal4 *UAS*-Wg/+

(c) *GMR*-Gal4 *UAS*-Dsh/+

(d) *UAS*-Arm/+; *GMR*-Gal4/+

(e) *GMR*-Gal4 *UAS*-Wg/*Df(3L)H99*

(f) *GMR*-Gal4 *UAS*-Dsh/*Df(3L)H99*

(g) *UAS*-Arm/+; *GMR*-Gal4/*Df(3L)H99*

(h) *GMR*-Gal4 *UAS*-Wg/*UAS*-Diap1

(i) *GMR*-Gal4 *UAS*-Dsh/*UAS*-Diap1

(j) *UAS*-Arm/+; *GMR*-Gal4/*UAS*-Diap1

(k) *UAS*-Dronc^{DN}/+; *GMR*-Gal4 *UAS*-Wg/+

(l) *UAS*-Dronc^{DN}/+; *GMR*-Gal4 *UAS*-Dsh/+

(m) *UAS*-Arm/*UAS*-Dronc^{DN}; *GMR*-Gal4/+

(n) *UAS-dronc-IR*/+; *GMR*-Gal4 *UAS*-Wg/+

(o) *UAS-dronc-IR*/+; *GMR*-Gal4 *UAS*-Dsh/+

(p) *UAS*-Arm/*UAS-dronc-IR*; *GMR*-Gal4/+

(q) *UAS-drice-IR*/+; *GMR*-Gal4 *UAS*-Wg/+

(r) *UAS-drice-IR*/+; *GMR*-Gal4 *UAS*-Dsh/+

(s) *UAS*-Arm/*UAS-drice-IR*; *GMR*-Gal4/+

(t) *GMR*-Gal4 *UAS*-Wg/*UAS-dcp-1-IR*

(u) *GMR*-Gal4 *UAS*-Dsh/*UAS-dcp-1-IR*

(v) *UAS*-Arm/+; *GMR*-Gal4/*UAS-dcp-1-IR*

Figure S6

(a) *GMR*-Gal4 *UAS*-Wg/+

(b) *GMR*-Gal4 *UAS*-Dsh /+

(c) *UAS*-Arm/+; *GMR*-Gal4/+

(d) *UAS*-Egr^{Regg1}/+; *GMR*-Gal4/+

(e) *GMR*-Gal4 *UAS*-Hep^{CA}/+

(f) *GMR*-Gal4 *UAS*-Dp53/+

(g) *GMR*-Gal4 *UAS*-Wg/*UAS*-P35

(h) *GMR*-Gal4 *UAS*-Dsh /*UAS*-P35

(i) *UAS*-Arm/+; *GMR*-Gal4/*UAS*-P35

(j) *UAS*-Egr^{Regg1}/+; *GMR*-Gal4/*UAS*-P35

(k) *GMR*-Gal4 *UAS*-Hep^{CA}/ *UAS*-P35

(l) *GMR*-Gal4 *UAS*-Dp53/+; *UAS*-P35/+

Figure S7

(a) *GMR*-Gal4 *UAS*-Wg/+

(b) *GMR*-Gal4 *UAS*-Dsh /+

(c) *UAS*-Arm/+; *GMR*-Gal4/+

(d) *GMR*-Gal4 *UAS*-Wg/*GUS*-Dp53^{259H}

(e) *GMR*-Gal4 *UAS*-Dsh/*GUS*-Dp53^{259H}

(f) *UAS*-Arm/+; *GMR*-Gal4/*GUS*-Dp53^{259H}

(g) *UAS*-Dp53^{H159N}/+; *GMR*-Gal4 *UAS*-Wg/+

(h) *UAS*-Dp53^{H159N}/+; *GMR*-Gal4 *UAS*-Dsh/+

(i) *UAS*-Arm/*UAS*-Dp53^{H159N}; *GMR*-Gal4/+

Figure S8

(a) *ptc*-Gal4/+; *puc*^{E69}/+

(b) *ptc*-Gal4 *UAS*-Hep/+; *puc*^{E69}/+

(c) *ptc*-Gal4 *UAS*-Hep/*UAS-wg-IR*; *puc*^{E69}/+

(d) *ptc*-Gal4 *UAS*-Hep/+; *puc*^{E69}/*UAS-pan-IR*

(e) *GMR*-Gal4/+; *puc*^{E69}/+

(f) *GMR*-Gal4/+; *puc*^{E69}/*UAS*-Hep^{CA}

(g) *GMR*-Gal4/+; *puc*^{E69}/*UAS*-Wg

(h) *GMR*-Gal4/+; *puc*^{E69}/*UAS*-Dsh

(i) *GMR*-Gal4/*UAS*-Arm; *puc*^{E69}/+

Figure S9

(a) *GMR*-Gal4 *UAS*-Wg/+

(b) *GMR*-Gal4 *UAS*-Dsh /+

(c) *UAS*-Arm/+; *GMR*-Gal4/+

(d) *GMR*-Gal4 *UAS*-Wg/*UAS*-Bsk^{DN}

(e) *GMR*-Gal4 *UAS*-Dsh/*UAS*-Bsk^{DN}

(f) *UAS*-Arm/+; *GMR*-Gal4/*UAS*-Bsk^{DN}

Figure S10

(a) *hs*-Flp; *act*>CD2>Gal4 *UAS*-GFP/+

(b) *hs*-Flp; *act*>CD2>Gal4 *UAS*-GFP/*UAS-puc-IR*

(c) *hs*-Flp; *act*>CD2>Gal4 *UAS*-GFP/*UAS-puc-IR; UAS-pan-IR*/+

Figure S11

(a) *ptc*-Gal4/+

(b) *ptc*-Gal4/+; *UAS-lgl-IR/*+

(c) *ptc*-Gal4/+; *UAS-lgl-IR/UAS*-Bsk^{DN}

(d) *ptc*-Gal4/+; *UAS-lgl-IR/UAS*-Puc

(e) *ptc*-Gal4/+; *UAS-lgl-IR/UAS-wg-IR*

(f) *ptc*-Gal4/+; *UAS-lgl-IR/UAS-dsh-IR*
